# Update on the Clinical Effect of Acupuncture Therapy in Patients with Gouty Arthritis: Systematic Review and Meta-Analysis

**DOI:** 10.1155/2016/9451670

**Published:** 2016-10-25

**Authors:** Wei-wei Lu, Jin-ming Zhang, Zheng-tao Lv, An-min Chen

**Affiliations:** Department of Orthopedics, Tongji Hospital, Tongji Medical College, Huazhong University of Science and Technology, Wuhan, Hubei 430030, China

## Abstract

*Objective*. The aim of this study is to evaluate the clinical efficacy and safety of acupuncture therapy in the treatment of acute gouty arthritis.* Methods*. A literature search of PubMed, EMBASE, ISI Web of Science, CENTRAL, and CNKI was conducted from the inception date of each database up to October 2015. Two investigators screened each article independently and were blinded to the findings of the other reviewer. Data was extracted according to the predetermined collection form. Meta-analysis was performed.* Results*. We analyzed data from 28 RCTs involving 2237 patients with gouty arthritis. Compared with conventional pharmacological treatments acupuncture was more effective in rendering patients free from symptoms after 24 hours, lowering serum urate, alleviating pain associated with gouty arthritis, and decreasing the ESR; regarding CRP, no statistically significant difference was found. In addition, the frequency of adverse events in acupuncture treatment was lower than that in control group.* Conclusion*. Based on the findings of our study, we cautiously suggest that acupuncture is an effective and safe therapy for patients with gouty arthritis. However, the potential beneficial effect of acupuncture might be overstated due to the methodological deficiency of included studies. High quality RCTs with larger scale are encouraged.

## 1. Introduction

Gouty arthritis is a disorder of purine metabolism and results from monosodium urate (MSU) crystal deposition in and around the joints caused by longstanding hyperuricaemia, which is defined as a serum uric acid (sUA) concentration exceeding the limit of sUA solubility (6.8 mg/dL) [1, 2]. This chronic inflammatory condition is mediated by MSU crystals and characterized by recurrent attacks of monoarthritis or polyarthritis [3]. Gouty arthritis is the most prevalent inflammatory arthritis in developed countries, especially in elder men; and the incidence was reported to increase over the past 20 years [4]. Quality of life is impaired during intercritical periods and during flares in addition to associated comorbidities such as obesity, diabetes mellitus, hypertension, hyperlipidaemia, and chronic renal disease [5–7]. Without proper treatment, acute gouty arthritis can progress into a chronic, deforming, and physically disabling disease through the development of tophi, joint destruction, and persistent pain [8, 9].

The American College of Rheumatology (ACR) and European League Against Rheumatism (EULAR) guidelines for the pharmacologic management of acute gouty arthritis include oral colchicine, nonsteroidal anti-inflammatory drugs (NSAIDs), and corticosteroids [10, 11]. However, the side effects associated with pharmacological agents restrict their long-term use. Some authors have pointed out that management of gouty arthritis is often suboptimal and that flares are inevitable, necessitating better strategies to handle attacks [12].

Complementary and alternative medicine (CAM) is widely advocated to face the increasing demand for nonpharmacological approaches. As a mainstream of CAM therapy, acupuncture based on traditional Chinese medicine (TCM) theory has been commonly used for the treatment of gouty arthritis in Chinese cultures. A previous systematic review had reported the clinical effect of acupuncture in relieving pain and decreasing serum uric acid, whereas only ten RCTs were included, the heterogeneity among studies was not discussed, and the security of acupuncture therapy was not confirmed [13]. Recently, new literatures to evaluate the efficacy and safety of acupuncture therapy for gouty arthritis are emerging. Therefore, an updated meta-analysis is required.

## 2. Methods

This systematic review was conducted in accordance with the Preferred Reporting Items for Systematic Reviews and Meta-Analyses (PRISMA) guidelines [14].

### 2.1. Search Strategy

A comprehensive literature search of PubMed, EMBASE, CENTRAL, ISI Web of Science, and CNKI was conducted. All the above databases were searched from their inception dates up to the latest issue (October 2015), without language restriction. Medical subject headings (MeSH) and free text words were combined to retrieve all the potential studies. MeSH were modified based on the specifications of each database. The following search strategy was used for the literature search in PubMed, CENTRAL, and ISI Web of Science: (“Gout” or Gout or gouts) and (“Acupuncture Therapy” or acupuncture or moxibustion or acupoint or acupressure or acustimulation). For CNKI, search terms were “Zhen” and “Tongfeng”. In addition, the bibliographies of relevant systematic reviews and clinical guidelines were manually searched. The reference section for each study was also searched.

### 2.2. Types of Participants

To be included in our systematic review, the enrolled subjects had to be diagnosed as gouty arthritis according to ascertained diagnostic criteria: European League Against Rheumatism (EULAR) criteria, the American Rheumatism Association (ARA) preliminary classification criteria for acute gout 1977 [15], Mexico 2010 [16], Netherland 2010 [17], or the Criteria of Diagnosis and Therapeutic Effect of Diseases and Syndromes in TCM issued by the State Administration of TCM [18]. No restrictions on race, age, and sex were imposed.

### 2.3. Interventions

Patients in experimental groups mainly received acupuncture therapy (including manual acupuncture and electroacupuncture), either alone or in combination with pharmacological treatment or TCM treatment, without differentiating different acupuncture techniques, acupoints selection, or needle materials. Patients in control groups were treated with western medicine (including colchicine, allopurinol, indomethacin, benzbromarone, celecoxib, probenecid, meloxicam, and ibuprofen); no specific types of drugs were imposed.

### 2.4. Outcome Measurements

The primary outcome measure was clinical effect and the frequency of adverse events in both groups; the secondary outcome measurements included clinical parameters associated with acute gouty arthritis, such as serum uric acid, pain intensity using visual analogue scale (VAS), erythrocyte sedimentation rate (ESR), and C-reactive protein (CRP).

### 2.5. Types of Publication

The included studies were required to be randomized controlled trials aiming to assess the efficacy of acupuncture therapy for gouty arthritis. Articles regarding animal experiments, reviews articles, case reports, or expert experience reports were excluded.

### 2.6. Data Extraction

Two investigators (Wei-wei Lu and Jin-ming Zhang) screened each article independently and were blinded to the findings of the other reviewer. According to the predetermined inclusion criteria, two reviewers performed strict screening to identify qualified articles independently, and they extracted data from these eligible articles using a standardized data collection form, which included first author, year of the publication, study design, baseline characteristics for participants in different groups, diagnostic criteria of gouty arthritis, interventions and control treatment, main outcome assessments, duration of treatments, and adverse events.

Any disagreement between the two reviewers was resolved through discussion until a consensus was reached. The third review author (Jin-ming Zhang) was consulted if a consensus could not be reached.

### 2.7. Quality Assessment

Cochrane Collaboration's tool was utilized to assess the risk of bias in the selected RCTs, which was based on seven items: random sequence generation, allocation concealment, blinding of participants and personnel, blinding of outcome assessment, incomplete outcome data, selective reporting, and other sources of bias [19]. Two reviewers assessed the risk of bias among studies independently; the results were compared afterwards. Disagreements regarding the risk of bias assessment were settled by discussion and consensus between reviewers.

### 2.8. Data Synthesis and Analysis

The enrolled participants were dichotomized into being cured and not cured to express the intervention effect. Odds ratio (OR) and the associated 95% confidence intervals (CIs) were calculated for clinical effect and frequency of adverse events. The mean difference (MD) for changes from baseline in the continuous variables was calculated using the same methodology. Random effect model was employed for meta-analysis, since the homogeneity of the included studies could not be guaranteed. The chi-squared test and the Higgins *I* ^2^ test were used to assess the heterogeneity among studies (*P* > 0.1 and *I* ^2^ indicate acceptable heterogeneity). Forest plot and funnel plot were generated via RevMan 5.3 (Copenhagen: the Nordic Cochrane Centre, the Cochrane Collaboration, 2014).

Metaregression was performed using Stata version 12.0 (StataCorp LP, USA) to find the possible source of heterogeneity, such as acupuncture type, treatment duration, and whether combined with other therapy. Begg's rank correlation test and Egger's linear regression test were used to evaluate the publication bias if the number of included studies was greater than ten.

## 3. Results

### 3.1. Literature Search Results

**Figure 1:**
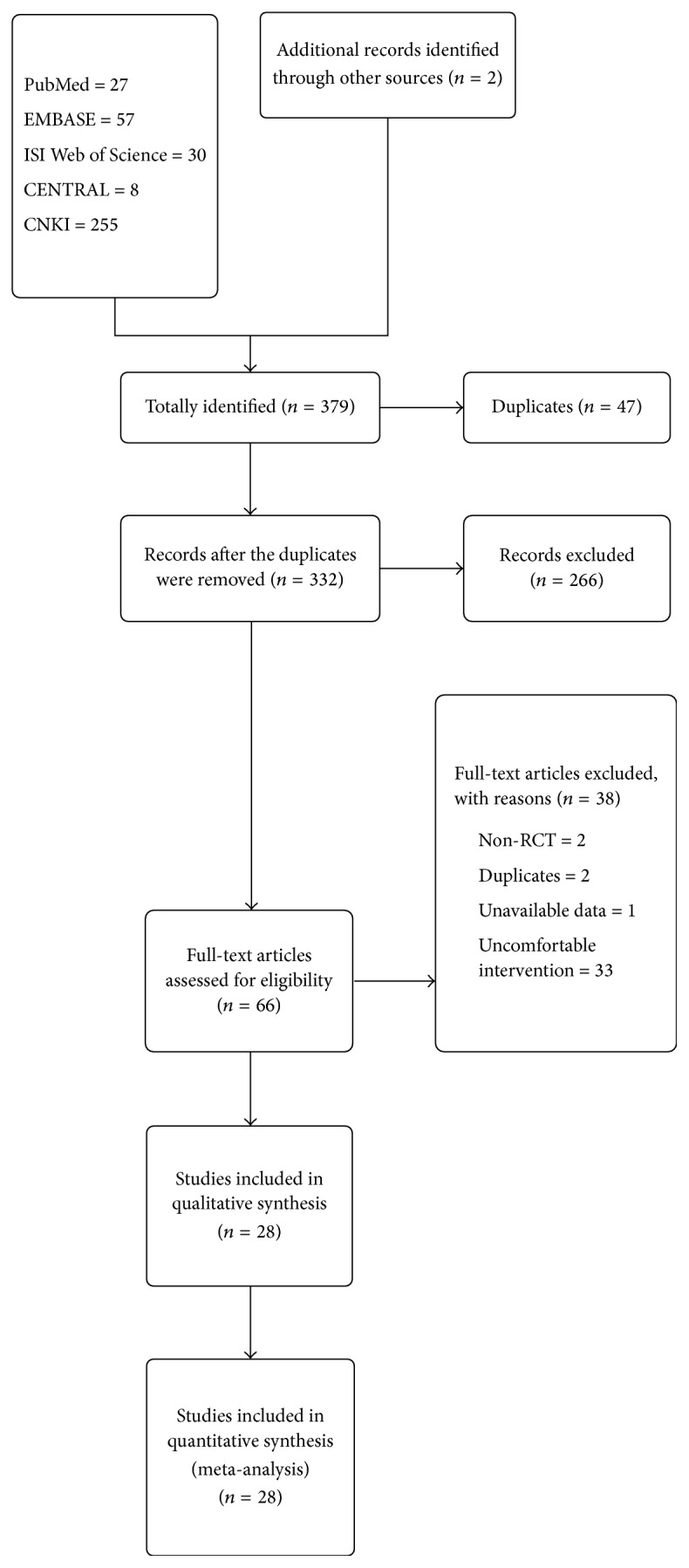
Flow diagram of the literature selection.

An initial search of RCTs yielded 379 potential literature citations, including 27 from PubMed, 8 from CENTRAL, 57 from EMBASE, 30 from ISI Web of Science, 255 from CNKI, and additional two records from other sources. 47 articles were deleted because they were duplicates for retrieving. According to the prespecified inclusion criteria, 66 potentially relevant studies were selected and retrieved for a full-text assessment after reading their titles and abstracts. Of the remaining 66 studies, two were deleted because they were non-RCTs, two were duplicates for publication, one study did not provide available data, and 33 studies employed uncomfortable intervention. A total of 28 studies [20–47] were deemed eligible for inclusion in this review. The literature screening process is presented in a flowchart in Figure 1.

### 3.2. Study Characteristics

The main characteristics of included studies are summarized in Table 1. The 28 studies included a total of 1174 patients in acupuncture group and 1063 patients in control group. Subjects were diagnosed as gouty arthritis according to either the criteria stipulated by US Rheumatology Association or the criteria stipulated by State Administration of TCM. The age of enrolled subjects ranged from 18 to 80 years. All the studies were conducted by Chinese investigators in a single center and published between 2002 and 2015. Clinical efficacy of acupuncture was evaluated by all studies, but only ten of them analyzed the safety in both groups.

Patients in acupuncture group received manual acupuncture or electroacupuncture; the acupoints selection was based on TCM meridian theory. The acupuncture was applied alone or in combination with other treatment, such as Chinese herbal medicine, acupoint injection, and local blocking therapy. Acupuncture therapy was administered daily or every two days, the needles were retained for 20 to 30 minutes for each session, and the treatment course ranged from 5 days to 28 days. The detailed information is listed in Table 2.

### 3.3. Quality Assessment

**Figure 2:**
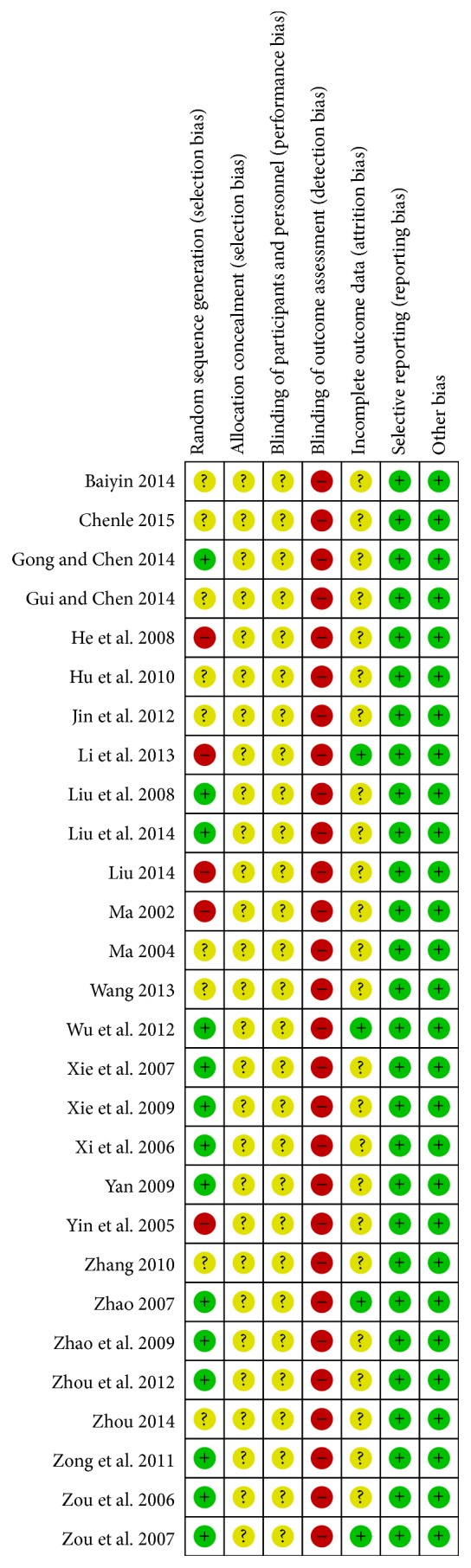
Risk of bias summary: review authors' judgements about each risk of bias item for each included study.

**Figure 3:**
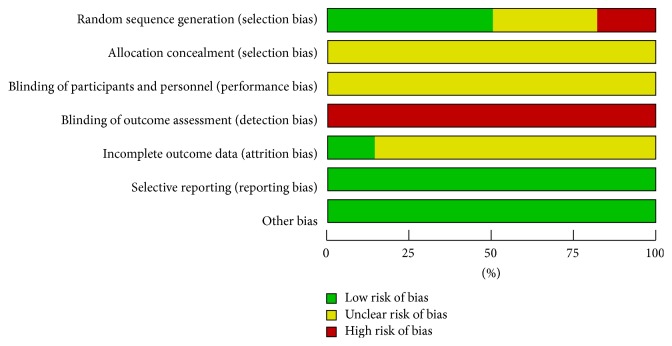
Risk of bias graph: review authors' judgements about each risk of bias item presented as percentages across all included studies.

The risk of bias among studies was assessed using Cochrane Collaboration's tool. All studies included the suggested randomization, half of the studies [22, 28, 29, 34–38, 41–43, 45–47] reported the method of random sequence generation, and five studies [24, 27, 30, 31, 39] were judged to high risk of bias because the patients were arranged according to their registration order. No study reported detail about allocation concealment. The blinding of outcome assessment was judged to unclear risk of bias because no studies mentioned blinding of outcome assessment. The blinding of participants and personnel was judged to high risk of bias as it was impossible to carry out in our included studies. When it comes to incomplete data, only four studies [27, 34, 41, 47] provided the number of dropouts and reason for withdrawal. All the studies reported the prespecified outcome measurements. Baseline similarities seemed to be achieved by each study: no statistical differences were detected in age, gender, or symptom duration. Five studies [24, 27, 30, 31, 39] were judged to high risk of bias while the other 23 were unclear risk of bias. Judgements about each risk of bias item for each included study were summarized in Figures 2 and 3.

### 3.4. Clinical Effect

**Figure 4:**
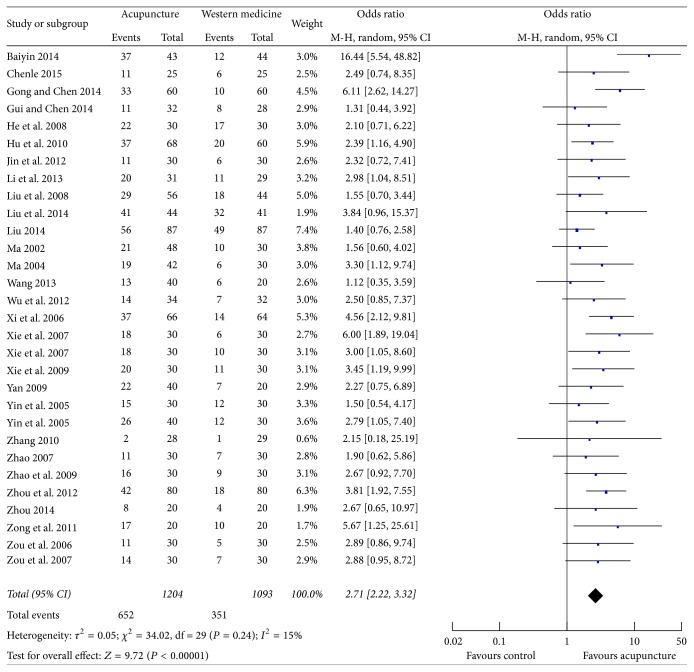
Forest plot of acupuncture therapy versus western medicine: clinical effect.

All the included studies employed clinical effect as outcome assessment; the therapeutic effect was evaluated in accordance with the Criteria of Diagnosis and Therapeutic Effect of Diseases and Syndromes in TCM issued by the State Administration of TCM in 1994 [18]. The patients were defined as complete resolution of acute attack when the joints swelling, pain, and redness disappeared with normal laboratory indices 24 hours after the treatment, improved when symptoms and signs alleviated with laboratory indices improved, or failed when the symptoms and laboratory indices did not have improvement. Thus, the patients in experimental and control groups were dichotomized as complete resolution and not completely resolved; OR and 95% CI in each study were calculated based upon the raw data. The combined effects of 28 individual studies showed that acupuncture therapy could further improve the clinical cure rate compared with western medicine (OR 2.71; 95% CI 2.22, 3.32; *P* < 0.00001); the results of heterogeneity test indicated no obvious heterogeneity (*P* = 0.24, *I* ^2^ = 15%) (Figure 4).

### 3.5. Uric Acid

**Figure 5:**
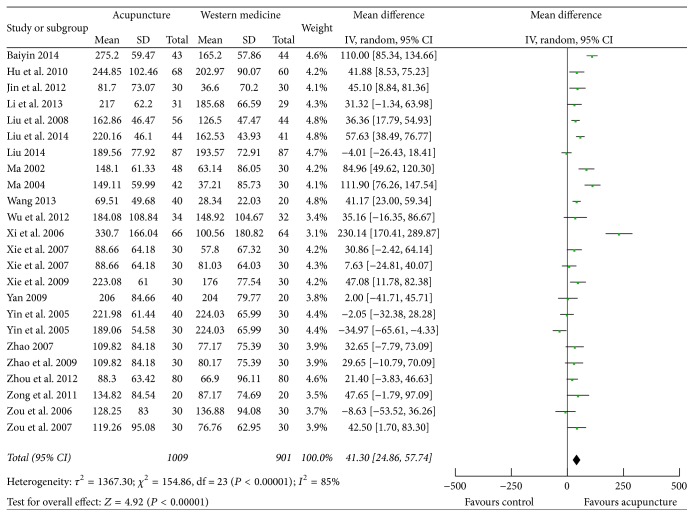
Forest plot of acupuncture therapy versus western medicine: uric acid.

Twenty-two studies [20, 25–39, 41–43, 45–47] measured blood uric acid as outcome; changes from baseline were calculated. Data extracted from 22 individual studies showed that heterogeneity existed (*P* < 0.00001, *I* ^2^ = 85.1%); random effects model was utilized for statistical analysis. Pooled data suggested that acupuncture therapy could further decrease uric acid than western pharmacological treatment (MD 41.30; 95% CI 24.86, 57.74; *P* < 0.00001) (Figure 5).

### 3.6. Pain Intensity (VAS Score)

**Figure 6:**
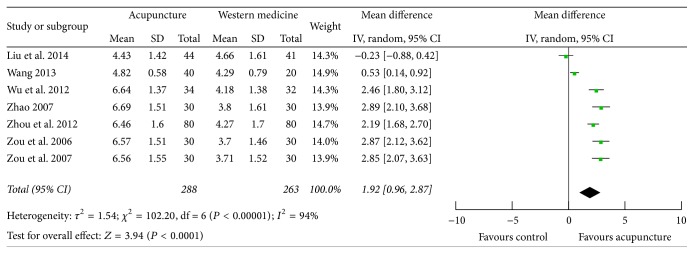
Forest plot of acupuncture therapy versus western medicine: pain intensity (VAS score).

Seven studies [29, 33, 34, 41, 43, 46, 47] that measured pain intensity using VAS score were identified. Data extracted showed obvious heterogeneity in the consistency of study results (*P* < 0.00001, *I* ^2^ = 94%); random effects model was employed. Among the seven studies, six found that acupuncture therapy could further improve the VAS score than pharmacological therapy, whereas Liu et al. reported an opposite result. The combination of results showed that acupuncture could further improve pain associated with acute gouty arthritis (MD 1.92; 95% CI 0.96, 2.87; *P* < 0.0001) (Figure 6).

### 3.7. ESR

**Figure 7:**
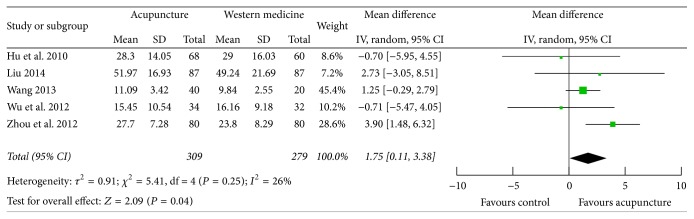
Forest plot of acupuncture therapy versus western medicine: ESR.

Five studies [25, 30, 33, 34, 43] reported on ESR; only one study found statistically significant difference regarding the decrease of ESR in acupuncture group and control group. The heterogeneity was acceptable (*P* = 0.25, *I* ^2^ = 26%); the pooled data showed that acupuncture therapy was better than western medicine in decreasing ESR (MD 1.75; 95% CI 0.11, 3.38; *P* = 0.04) (Figure 7).

### 3.8. CRP

**Figure 8:**
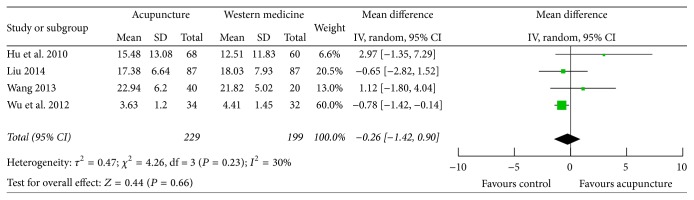
Forest plot of acupuncture therapy versus western medicine: CRP.

Four studies [25, 30, 33, 34] measured CRP as outcome; statistically significant difference was detected in only one study. No obvious heterogeneity was found (*P* = 0.23, *I* ^2^ = 30%); the combined data showed no significant difference in decreasing CRP between acupuncture and control therapy (MD −0.26; 95% CI −1.42, 0.90; *P* = 0.66) (Figure 8).

### 3.9. Adverse Events

**Figure 9:**
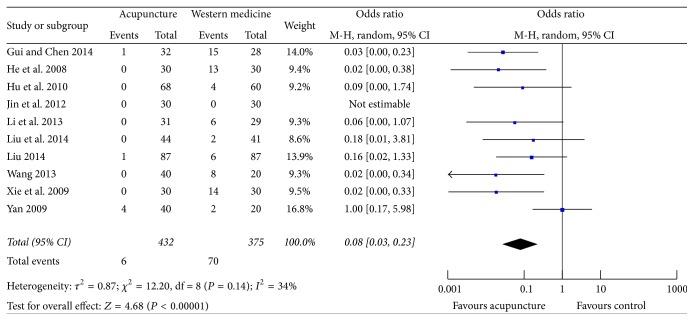
Forest plot of acupuncture therapy versus western medicine: adverse events.

Among the included studies, ten [23–27, 29, 30, 33, 37, 38] reported adverse events associated with acupuncture or western medicine. The reported adverse events mainly included gastrointestinal tract reaction, central nervous system reaction, leukopenia, skin rash, and fainting during acupuncture treatment. Except two studies [26, 38], eight studies found a higher prevalence of adverse events in the control group than that in the acupuncture group. The pooled OR showed a statistically significant lower risk of adverse events in acupuncture group when compared with western medicine (OR 0.08, 95% CI 0.03, 0.23; *I* ^2^ = 34%) (Figure 9).

### 3.10. Metaregression

Metaregression was conducted by residual (restricted) maximum likelihood (REML) with Knapp-Hartung modification. Three possible factors that may contribute to heterogeneity among studies were tested: acupuncture type (manual acupuncture or electroacupuncture), combined therapy (whether the patients were treated with acupuncture alone or combined with other treatment), and duration of treatment (within a week or longer than a week). The results of metaregression were listed in Table 3. Combined therapy and duration of treatment were not statistically correlated with the heterogeneity in uric acid or VAS score. Acupuncture type administered could explain 20.20% and 23.55% of the heterogeneity in uric acid and VAS score, respectively. However, the correspondence between acupuncture type and heterogeneity within VAS score did not reach a statistical difference.

### 3.11. Publication Bias

**Figure 10:**
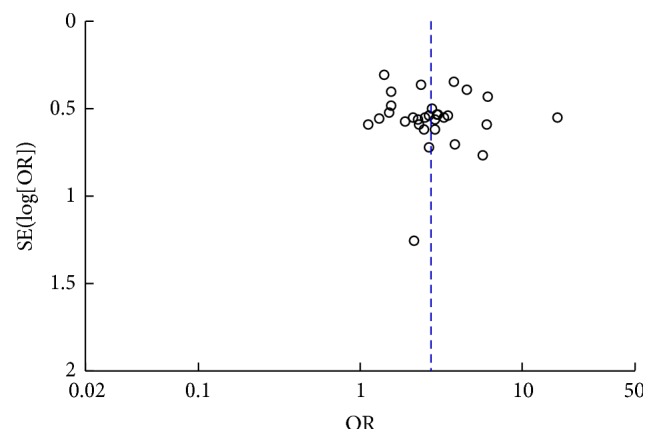
Funnel plot of acupuncture therapy versus western medicine: clinical effect.

**Figure 11:**
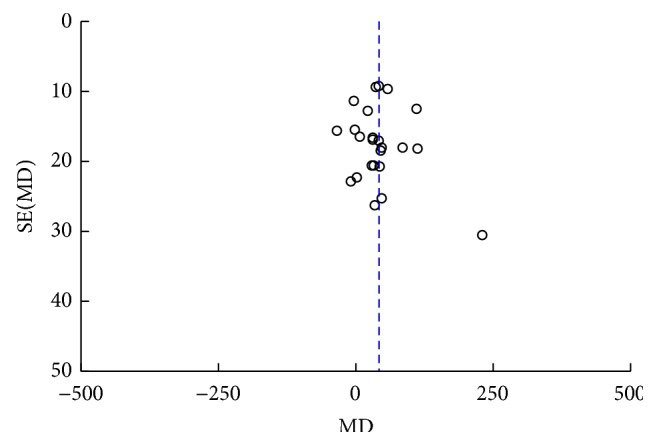
Funnel plot of acupuncture therapy versus western medicine: uric acid.

Publication biases were presented by funnel plots (Figures 10 and 11), and the resulting graphs show no obvious asymmetry for clinical effect and uric acid. Begg's test (clinical effect: *z* = 0.86, *P* = 0.392; uric acid: *z* = 0.94, *P* = 0.346) and Egger's test (clinical effect: *t* = 1.00, *P* = 0.325; uric acid: *t* = 0.46, *P* = 0.650) also indicated no statistically significant publication bias.

## 4. Discussion

Our current study analyzed data from 28 RCTs involving 2237 patients that aimed to assess the therapeutic effect and safety of acupuncture for gouty arthritis. Based on the findings of our study, acupuncture could further improve the clinical effective rate and decrease uric acid and VAS score when compared with western medicine. The risk of adverse events was significantly lower in acupuncture group. Regarding the decrease in CRP and ESR, the results remain debatable.

The application of different acupuncture modalities by different investigators can greatly affect curative effect of acupuncture therapy [48]. The acupuncture procedures should be performed according to syndromes differentiation based on TCM theory. In the selected studies, acupuncture intervention was administered alone [22, 31, 32, 35–37, 41, 46] or in combination with other therapies, which included Chinese herbal medicine [20, 21, 23, 25–27, 29, 30, 33, 44], acupoint injection [24, 47], local blocking therapy [28], bloodletting [34, 38, 42], infrared irradiation [43], moxa-moxibustion [45], and western medicine [39, 40]. Regardless of the type of the additional therapy, acupuncture therapy was mainly received in experimental groups. The acupoint selection was inconsistent among included studies; nevertheless, Sp6, St36, and Ashi point were the most commonly used acupoint. In our present study, data was combined without differentiating acupoint selection and acupuncture techniques. Thus, the results indicate an overall clinical efficacy and definite conclusion could not be drawn.

Gouty arthritis is one of the most common indications for which patients seek complementary and alternative medicine treatment, even though the use of CAM was relatively low, compared with reported rates of between 28% and 90% in patients with rheumatoid arthritis (RA) and more than 80% in those with osteoarthritis (OA) [49, 50]. The goal of therapy in an acute gout attack is prompt and safe termination of pain and inflammation [51]. Our results suggest that acupuncture therapy could further improve pain when compared with western medicine, but, in terms of inflammation attenuation, the results remain debatable.

When treating gouty arthritis one needs to treat acute attacks and lower excess stores of uric acid to achieve dissolution of monosodium urate crystals through a long-term reduction of serum uric acid concentrations far beyond the threshold for saturation of urate and provide prophylaxis to prevent acute flares [52]. In addition to the improvement in pain-relief and clinical effect, acupuncture therapy could further decrease uric acid, compared with conventional western pharmacological therapy. The underlying mechanism of this urate-lowering effect was still unclear, which needs to be further investigated. To analyze the possible source of heterogeneity within these studies, metaregression was performed. The type of acupuncture therapy was proved to be significantly correlated with the heterogeneity; the evidence thus suggests that the urate-lowering effects of manual acupuncture and electroacupuncture are somewhat different. No conclusions can be drawn as to which acupuncture type is superior for patients with gouty arthritis.

A previous systematic review [13] consisting of ten RCTs reported that acupuncture alone was more effective in the improvement of pain and uric acid when compared with standard western medicine. However, only ten studies were identified to be eligible; only VAS score and uric acid were analyzed as outcome. No data about the safety of acupuncture therapy was provided. In addition, two records listed in Figure 1A of the previous systematic review were miscalculated, although this would not influence the final conclusion drawn by their work [13]. Based on five required domains (study limitations, consistency, directness, precision, and publication bias), Shekelle and colleagues assessed the strength of evidence for the conclusions drawn by the aforementioned systematic review [53]. The strength of evidence for conclusions was judged to be insufficient to support or refute the effectiveness of acupuncture on symptomatic outcomes, partially due to the unreported publication bias. Our meta-analysis managed to summarize all published RCTs to compare the clinical effect and safety of acupuncture with those of western medicine. 28 RCTs were identified; clinical effect, VAS score, uric acid, ESR, CRP, and adverse events were combined; risk of bias was independently assessed by two experienced reviewers using Cochrane's tool. The results of metaregression showed a significant correlation between acupuncture type and heterogeneity in uric acid; publication bias assessment indicates no obvious publication bias. Regarding the safety of acupuncture, which is an obvious advantage of CAM therapies, the overall incidence of adverse events in acupuncture groups was significantly lower than that in western medicine group.

There are several limitations in our study. First, the methodological qualities of included studies were judged to be poor; details about allocation concealment were not described by any study, which might limit the value of conclusion about the clinical efficacy and safety of acupuncture. Second, all included studies utilized western medicine as control treatment, making the blinding of participants impossible. A sham acupuncture control is preferable, as opposed to medication or no intervention. Also, the majority of selected studies were written in Chinese, which limits the dissemination of scientific researches on acupuncture. Future studies within western context are required. Third, financial considerations are important for consumers choosing CAM options as expensive treatments are generally avoided and cost is a frequent reason to stop treatment [54]. In Chinese culture, the safety and cost-effectiveness of acupuncture therapy could assure the compliance of patients. Considering participants' opinions, cost-effectiveness of acupuncture also meant its efficacy. However, only ten studies recorded adverse events associated with acupuncture and routine care; no study included cost-effectiveness assessment. Last, according to the ACR guideline, choice of pharmacologic agents should be based upon number of joints involved and pain intensity; the established pharmacologic ULT should be continued without interruption. However, some of our included studies only administered first-line therapy options (NSAIDs, corticosteroids, and colchicine); ULT therapy during acute attack was stopped; some studies only used ULT, without using first-line pharmacologic options. This would lead to an exaggeration of conclusions; thus, the effect of intervention should be interpreted cautiously.

In summary, the findings of our current study suggest that acupuncture is better in improving clinical effective rate, decreasing uric acid, and VAS score compared with western medicine. The overall incidence of adverse events in acupuncture group was lower than that in western medicine group. Due to the methodological deficiency of included trials, acupuncture therapy could not be guaranteed as standard CAM treatment. Additional RCTs with rigorous design and larger sample size are encouraged.

## Figures and Tables

**Table 1:** Characteristics of RCTs identified in the literature search.

Study	Population (E/C)	Age (mean or range)	Diagnostic criteria	Outcome measurements	Adverse events
Baiyin 2014 [20]	43/44	E: 27–56; C: 28–54	ARA 1977	Clinical effect, uric acid	NR
Chenle 2015 [21]	25/25	E: 25–58; C: 23–60	ARA 1977	Clinical effect	NR
Gong and Chen 2014 [22]	60/60	NR	ARA 1977	Clinical effect	NR
Gui and Chen 2014 [23]	32/28	E: 28–65; C: 26–62	SATCM 1994	Clinical effect	Yes
He et al. 2008 [24]	30/30	E: 28–67; C: 30–65	SATCM 1994	Clinical effect	Yes
Hu et al. 2010 [25]	68/60	E: 45.42 ± 11.46; C: 46.74 ± 11.22	ARA 1977	Clinical effect, uric acid, CRP, ESR	Yes
Jin et al. 2012 [26]	30/30	E: 23-67; C: 25–66	ARA 1977	Clinical effect, uric acid	Yes
Li et al. 2013 [27]	31/29	E: 49.67 ± 19.3; C: 46.52 ± 10.29	ARA 1977	Clinical effect, uric acid	Yes
Liu et al. 2008 [28]	56/44	E: 34–70: C: 33–74	SATCM 1994	Clinical effect, uric acid	NR
Liu et al. 2014 [29]	44/41	E: 20–50; C: 18–52	ARA 1977	Clinical effect, uric acid, VAS score	Yes
Liu 2014 [30]	87/87	E: 44.1; C: 43.4	ARA 1977	Clinical effect, uric acid, CRP, ESR	Yes
Ma 2002 [31]	48/30	E: 31–78; C: 29–72	ARA 1977	Clinical effect, uric acid	NR
Ma 2004 [32]	42/30	E: 31–78; C: 29–72	ARA 1977	Clinical effect, uric acid	NR
Wang 2013 [33]	40/20	E: 23–70; C: 23–70	ARA 1977	Clinical effect, ESR, CRP, uric acid	Yes
Wu et al. 2012 [34]	34/32	E: 32–60; C: 33–63	SATCM 1994	Clinical effect, uric acid, CRP, ESR, VAS score	NR
Xi et al. 2006 [35]	66/64	E: 50.1 ± 5.2; C: 45.1 ± 6.1	ARA 1977	Clinical effect, uric acid, VAS score	NR
Xie et al. 2007 [36]	30/30/30	E: 40–70; C1: 42–69; C2: 43–71	ARA 1977	Clinical effect, uric acid	NR
Xie et al. 2009 [37]	30/30	E: 32–65; C: 40–67	SATCM 1994	Clinical effect, uric acid	Yes
Yan 2009 [38]	40/20	E: 35–75; C: 38–76	ARA 1977	Clinical effect, uric acid	Yes
Yin et al. 2005 [39]	40/30/30	E1: 36–72: E2: 31–69: C: 34–76	SATCM 1994	Clinical effect, uric acid	NR
Zhang 2010 [40]	28/29	E: 38–79; C: 29–80	SATCM 1994	Clinical effect	NR
Zhao 2007 [41]	30/30	E: 31–68; C: 30–70	SATCM 1994	Clinical effect, uric acid, VAS score	NR
Zhao et al. 2009 [42]	30/30	E: 33–70; C: 32–71	ARA 1977	Clinical effect, uric acid	NR
Zhou et al. 2012 [43]	80/80	E: 36–65; C: 37–64	SATCM 1994	Clinical effect, uric acid, VAS score, ESR	NR
Zhou 2014 [44]	20/20	E: 18–48; C: 17–50	SATCM 1994	Clinical effect	NR
Zong et al. 2011 [45]	20/20	E: 34–72; C: 30–70	ARA 1977	Clinical effect, uric acid	NR
Zou et al. 2006 [46]	30/30/30	E1: 32–70; E2: 31–72; C: 35–71	SATCM 1994	Clinical effect, uric acid, VAS score	NR
Zou et al. 2007 [47]	30/30	E: 32–70; C: 31–72	SATCM 1994	Clinical effect, uric acid, VAS score	NR

Note. E: experiment; C: control; NR: not reported; CRP: C-reactive protein; ESR: erythrocyte sedimentation rate; TCM: traditional Chinese medicine; VAS: visual analogue scale; ARA 1977: the American Rheumatism Association preliminary classification criteria for acute gout 1977; SATCM 1994: the Criteria of Diagnosis and Therapeutic Effect of Diseases and Syndromes in Traditional Chinese Medicine issued by the State Administration of Traditional Chinese Medicine.

**Table 2:** Details of intervention in acupuncture groups and control groups.

Study	Acupuncture intervention	Control	Duration
Baiyin 2014 [20]	MA (Ashi point) 25 min, once a day, plus Chinese herbal medicine	Colchicine 0.5 mg twice a day, allopurinol 50 mg twice a day	15 days
Chenle 2015 [21]	MA (Ashi point, Gb34, Sp10, St35, EX-LE4, S34, EX13) 25 min, once a day, plus Chinese herbal medicine	Colchicine 0.5 mg twice a day, allopurinol 50 mg twice a day	15 days
Gong and Chen 2014 [22]	MA (Ashi point), every other day	Allopurinol 50 mg 3 times a day	20 days
Gui and Chen 2014 [23]	MA (Ashi point, Sp9, St36, Sp6, Li11, Gv14), once a day, plus Chinese herbal medicine	Allopurinol 100 mg 3 times a day, colchicine 0.5 mg/hour	10 days
He et al. 2008 [24]	EA (Ashi point, St36, Sp6, Ki3, Sp4) 20 min, once a day, plus acupoint injection	Colchicine 0.5 mg 3 times a day, indometacin 25 mg 3 times a day	10 days
Hu et al. 2010 [25]	MA (Gb11, Cv6, Ki3, B40) 30 min, once a day, plus Chinese herbal medicine	Indometacin 25 mg 3 times a day, allopurinol 100 mg 3 times a day	21 days
Jin et al. 2012 [26]	MA (Sp6, Sp9, Li11, S34, Sp10, H3, L15), once a day, plus Chinese herbal medicine	Allopurinol 100 mg twice a day	7 days
Li et al. 2013 [27]	MA (Sp6, St36, Sp9, St40, Sp10, Li11, Li4, Lr3) 15 min, once a day, plus Chinese herbal medicine	Colchicine 0.5 mg 3 times a day	21 days
Liu et al. 2008 [28]	EA (Ashi point, Sp1, Lr3, Sp6, St40, Sp9, Gb34) 30 min, once a day, plus local blocking therapy	Allopurinol 100 mg 3 times a day, indometacin 25 mg 3 times a day	7 days
Liu et al. 2014 [29]	MA (surrounded the diseased region), once a day, plus external Chinese herbal medicine	Colchicine 0.5 mg twice a day, celecoxib 200 mg twice a day	7 days
Liu 2014 [30]	MA (Sp10, Sp6, St36, St40, Sp9, Li11, Li4, Lr3) 30 min, once a day, plus Chinese herbal medicine	Benzbromarone 50 mg once a day	14 days
Ma 2002 [31]	MA (Sp6, Ki3, Sp10, Li11) 30 min, once a day	Indometacin 25 mg 3 times a day, allopurinol 100 mg 2-3 times a day	28 days
Ma 2004 [32]	MA (Bl23, Bl22, Cv3, Cv4, Sp10, Sp6, Ki3) 30 min, once a day	Allopurinol 100 mg 2-3 times a day	28 days
Wang 2013 [33]	MA (Ashi point, Ki3, Sp9, B60, Li11, Li4, Lr3) 20 min, once a day, plus Chinese herbal medicine	Colchicine 0.5 mg 3 times a day	14 days
Wu et al. 2012 [34]	EA 2 Hz (St36, St40, Ashi point) 30 min, once a day, plus bloodletting	Colchicine 0.5 mg 3 times a day, allopurinol 100 mg 3 times a day	6 days
Xi et al. 2006 [35]	MA (Ashi point, Sp6, Sp10, Sp9, Li4, Li11) 30 min, once a day	Meloxicam 7.5 mg once a day	20 days
Xie et al. 2007 [36]	EA (Sp6, Sp9, St40) 30 min, once a day	Allopurinol 100 mg 3 times a day, probenecid 250 mg 3 times a day	10 days
Xie et al. 2009 [37]	MA (surrounded the diseased region) 30 min, once a day	Allopurinol 100 mg 3 times a day, indometacin 25 mg 3 times a day	15 days
Yan 2009 [38]	MA (Ashi point, Ki3, Sp6, St36, B60, St40, Lr3) 30 min, once a day, plus bloodletting	Allopurinol 100 mg twice a day	14 days
Yin et al. 2005 [39]	EA (St36, St40, Ashi point) 30 min, once a day; EA plus western medicine	Allopurinol 100 mg 3 times a day, indometacin 25 mg 3 times a day	6 days
Zhang 2010 [40]	MA 30 min, once a day, plus western medicine	Indometacin 25 mg 3 times a day	6 days
Zhao 2007 [41]	MA (St36, Sp6, Ashi point), once a day	Indometacin 25 mg 3 times a day, allopurinol 100 mg 3 times a day	6 days
Zhao et al. 2009 [42]	EA (Ashi point, Li4, St36, Gb34, Sp10, B60, Ki3) 30 min, once a day, plus bloodletting	Probenecid 250 mg twice a day	10 days
Zhou et al. 2012 [43]	MA (Sp10, St36, Sp6, Li11, Gb34) 30 min, once a day, plus infrared irradiation	Indometacin 25 mg 3 times a day	5 days
Zhou 2014 [44]	MA (Ashi point, Li11, Sp10, Sp6, Gv14, Lr3, St36) 30 min, once a day, plus Chinese herbal medicine	Ibuprofen 300 mg twice a day	7 days
Zong et al. 2011 [45]	MA (Ashi point, Sp9, St36, Sp6, Li11, Li4, S34) 30 min, once a day, plus moxa-moxibustion	Allopurinol 100 mg 3 times a day, indometacin 25 mg 3 times a day	6 days
Zou et al. 2006 [46]	EA 100 Hz (St36, Sp6) 30 min, once a day; EA 2 Hz (St36, Sp6) 30 min, once a day	Allopurinol 100 mg 3 times a day, indometacin 25 mg 3 times a day	6 days
Zou et al. 2007 [47]	EA 2 Hz (St36, Sp6) 30 min, every other day, plus acupoint injection	Allopurinol 100 mg 3 times a day, indometacin 25 mg 3 times a day	6 days

Note. EA: electroacupuncture; MA: manual acupuncture; Ashi point: pain spot; Sp10: Xuehai; Sp9: Yinlingquan; St36: zusanli; Ki3: taixi; Gb11: touqiaoyin; Cv6: qihai; Sp6: sanyinjiao; Li11: quchi; Lr3: taichong; Bl23: shenyu; Bl22: sanjiaoyu; Cv3: zhongji; St40: fenglong; B60: Kunlun; St35: dubi; EX-LE4: neixiyan; S34: liangqiu; EX13: heding; Gv14:dazhui; Sp4: gongsun; B40: weizhong; H3: shaohai; L15: chize; Li4: hegu; Cv4: guanyuan; B12: fengshi.

**Table 3:** Metaregression of basic characteristics of trials and improvement of uric acid and VAS score.

Outcome	Number of RCTs	Factor tested	*t*	*P*	Adjusted *R* ^2^
Uric acid	22	Acupuncture type	2.21	0.038	20.20%
		Combined therapy	0.35	0.730	−6.31%
		Duration of treatment	1.58	0.127	7.67%
VAS score	7	Acupuncture type	−1.62	0.165	23.55%
		Combined therapy	1.32	0.243	12.20%
		Duration of treatment	−1.27	0.259	10.11%

Note. RCT: randomized controlled trial; VAS: visual analogue scale.
